# Complete mitogenome of the red squirrel *Sciurus vulgaris* (Sciuridae) from Korea

**DOI:** 10.1080/23802359.2017.1365646

**Published:** 2017-08-17

**Authors:** Hye Ri Kim, Ji Young Kim, Eui Kyeong Kim, Hyun Ju Kim, Yung Chul Park

**Affiliations:** a Ecosystem Research Division, National Park Research Institute, Korea National Park Service, Wonju, Republic of Korea;; b Division of Forest Science, College of Forest & Environmental Sciences, Kangwon National University, Chuncheon, Republic of Korea;; c International Technology Cooperation Center, Rural Development Administration, Wanju, Republic of Korea

**Keywords:** Mitogenome, red squirrel, *Sciurus vulgaris*, Sciuridae

## Abstract

The mitogenome of the Korean *S. vulgaris* is a circular molecule of 16,511 bp, consisting of a control region and a conserved set of 37 genes containing 13 protein-coding genes (PCGs), 22 tRNA genes and 2 rRNA genes (*12S rRNA* and *16S rRNA*). The mitogenome of the Korean *S. vulgaris* is AT-biased, with a nucleotide composition of 32.0% A, 30.9% T, 12.6% G and 24.5% C. The phylogenetic analysis revealed that the Korean red squirrel *S. vulgaris* is well grouped with the European red squirrel *S. vulgaris* and formed a sister clade to the Old World flying squirrels of the genus *Pteromys.*

The red squirrels of *Sciurus vulgaris* (Sciuridae), also known as the Eurasian red squirrel, are distributed across the forests of the Palearctic from the western Europe and the UK eastward to the pacific coast of East Asia (Oshida et al. 2009; Liu et al. 2014). In South Korea, the red squirrels are widely distributed across coniferous forests and temperate broadleaf woodland. We sequenced and characterized the complete mitogenome of the Korean *S. vulgaris*. A tissue sample for genomic DNA extraction was collected from a red squirrel individual caught around an agricultural region in Odaesan National Park (N37 43 23.4, E128 36 03.8), South Korea. The voucher specimen (SCSCVU-1) was deposited in the National Park Research Institute, Korea National Park Service. Genomic DNA extraction, PCR and gene annotation were conducted according to the previous studies (Yoon et al. 2013; Jeon and Park 2015; Rahman et al. 2016). A previously published mitogenome of the European *S. vulgaris* (AJ238588) was used as reference for gene annotation and primer design for PCR amplification of the Korean *S. vulgaris* mitogenome. Phylogenetic tree was constructed using maximum-likelihood (ML) procedures implemented in MEGA6 (Tamura et al. 2013).

The complete mitogenome (KU962990) of the Korean *S. vulgaris* contains 16,511 bp in length, which consists of a control region (CR) and a conserved set of 37 vertebrate mitochondrial genes including 13 PCGs, 22 tRNA genes and 2 rRNA genes (*12S rRNA* and *16S rRNA*). The order and orientation of these genes are identical to those of other mammals (Kim and Park 2012; Yoon et al. 2013; Nam et al. 2015).

The whole mitogenome of the Korean *S. vulgaris* is AT-biased, with a nucleotide composition of 32.0% A, 30.9% T, 12.6% G and 24.5% C. Total length of the 13 mitochondrial PCGs of the Korean *S. vulgaris* is 11,367 bp long, with the exclusion of stop codons, which encode 3789 amino acids. Lengths of two rRNA genes (12*s rRNA* and 16s *rRNA*) were 967 bp and 1579 bp long, respectively. As rRNA genes in other mammal mitogenomes (Kim and Park 2012; Yoon et al. 2013; Nam et al. 2015), *12S rRNA* and *16S rRNAs* of the Korean *S. vulgaris* mitogenome are located between *tRNA^Phe^* and *tRNA^Leu(UUR)^* and separated by *tRNA^Val^*. There are a total of 22 tRNA genes for transferring 20 amino acids, ranging in size from 59 bp (*tRNA^Ser(AGY)^*) to 75 bp (*tRNA^Leu(UUR)^*). The tRNA genes include two leucine-tRNA genes (*tRNA^Leu(UUR)^* and *tRNA^Leu(CUN)^*) and two serine-tRNA genes (*tRNA^Ser(AGY)^*and *tRNA^Ser(UCN)^*). The mitochondrial *O_R_*is 32 bp long and is located between *tRNA^Asn^* and *tRNA^Cys^* in the WANCY region, which consists of a cluster of five tRNA genes (*tRNA^Trp^*, *tRNA^Ala^*, *tRNA^Asn^*, *tRNA^Cys^* and *tRNA^Tyr^*). Mitochondrial CR, which is located between *tRNA^Pro^* and *tRNA^Phe^*, is 1058 bp long.

The phylogenetic analysis revealed that the Korean red squirrel *S. vulgaris* is well grouped with the European red squirrel *S. vulgaris* and formed a sister clade to the Old World flying squirrels of the genus *Pteromys* (Figure 1).

**Figure 1.**
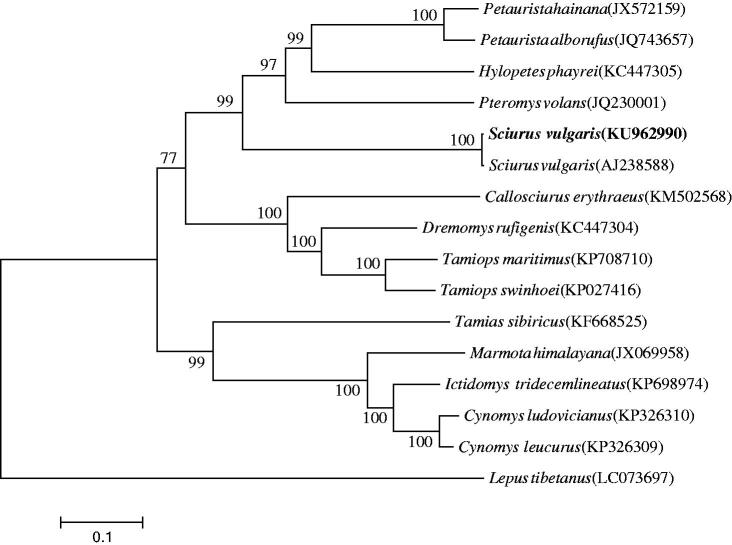
The phylogenetic relationship of *S. vulgaris* and its allied species inferred from maximum-likelihood analysis based on mitogenome sequences. The ML tree was generated using the GTR + G + I model, and the robustness of the tree was tested with 1000 bootstrap. The numbers on the branches indicate bootstrap values.
